# ﻿New records of rove beetles from the Province of Quebec, and additional provincial records in Canada (Coleoptera, Staphylinidae)

**DOI:** 10.3897/zookeys.1196.118698

**Published:** 2024-04-01

**Authors:** Nicolas Bédard, Adam Brunke, Pierrick Bloin, Ludovic Leclerc

**Affiliations:** 1 Natural Resources Canada, Canadian Forestry Service, Laurentian Forestry Centre, 1055, rue du P.E.P.S., C. P. 10380, Quebec, QC G1V 4C7, Canada Natural Resources Canada Quebec Canada; 2 Canadian National Collection of Insects, Arachnids and Nematodes, Agriculture and Agri-Food Canada, 960 Carling Avenue, Ottawa, ON, K1A 0C6, Canada Canadian National Collection of Insects, Arachnids and Nematodes, Agriculture and Agri-Food Canada Ottawa Canada; 3 Laval University, 12325, rue de l’Université, Quebec, QC G1V 0A6, Canada Laval University Quebec Canada

**Keywords:** Euaesthetinae, Omaliinae, Paederinae, Proteininae, Pselaphinae, Scaphidiinae, Staphylininae, Steninae, Tachyporinae

## Abstract

We newly report 25 provincial records of rove beetles (Coleoptera: Staphylinidae) from the province of Quebec from the following subfamilies: Steninae (1), Euaesthetinae (1), Omaliinae (2), Oxyporinae (1), Paederinae (1), Proteininae (1), Pselaphinae (2), Scaphidiinae (2), Scydmaeninae (2), Staphylininae (11) and Tachyporinae (1). Among these, two species are also reported for the first time from Ontario, two from Nova Scotia, and five are new Canadian records. We also report the first supporting data for *Suniusmelanocephalus* (Fabricius, 1792) and *Scopaeusminutus* Erichson, 1840 for Quebec, and of *Arpediumschwarzi* Fauvel, 1878, *Phyllodrepapunctiventris* (Fauvel, 1878), and *Sepedophilusbasalis* (Erichson, 1839) for Ontario. Specimen data and diagnoses are provided for each species, as well as references for identification where available.

## ﻿Introduction

The rove beetles (Staphylinidae) are one of the most speciose insect groups, with more than 66,000 described species (Newton 2022) and many more to discover. However, faunistic knowledge and a precise inventory are still lacking in most parts of the world. Many recent works (e.g., Brunke and Marshall 2011; Brunke et al. 2011, 2012a, 2021; Webster and Demerchant 2012a, 2012b, Webster et al. 2012a, 2012b, 2012c, 2012d, 2012e, 2012f, 2016; Brunke 2016; Klimaszewski et al. 2016, 2017, 2020, 2021) have documented and greatly expanded the knowledge on species diversity in Canada. In the latest checklist of the beetles of Canada and Alaska (Bousquet et al. 2013), there were 769 species of rove beetles known from the province of Quebec. Since then, this number has significantly increased, whether through the descriptions of new species, or by more extensive inventories resulting in the discovery of broader distributions for described species. In recent years, molecular-based approaches to faunistics have provided several important additions to the Staphylinidae of Canada and underlined the necessity for a deeper and more extensive taxonomic study of Nearctic beetles (Hebert et al. 2016; Pentinsaari et al. 2019; Brunke et al. 2021). Despite the enormous changes to the fauna of Canada and Quebec since Bousquet et al. (2013), many staphylinid groups remain in the same or similar state of knowledge as reported in Brunke et al. (2012b). The small size of most species, their great diversity, and the lack of easily observed, external diagnostic characters in many groups have traditionally made these beetles less attractive for collectors, but their ubiquity and diversity of ecological roles make them extremely useful tools for ecological and conservation studies (Pohl et al. 2008).

Following the examples set by the above cited works, and in order to better document species in northeastern Canada, the authors have increased their efforts in the last few years to collect and identify many specimens of rove beetles from various locations in the province of Quebec. This collecting effort, in conjunction with intensive curational work in some major collections, has led to the discovery of several unrecorded species in the province and elsewhere in Canada. We report 25 new records of Staphylinidae in Quebec (excluding the Aleocharinae, which will be treated in a separate paper), with two additions to the Ontario fauna and two from Nova Scotia. We also provide images for poorly known species that have not been clearly illustrated previously in the North American taxonomic literature.

## ﻿Materials and methods

Acronyms of collections referred to in this publication are as follows:

**CCC** Claude Chantal Insect collection (private collection), Varenne, Quebec, Canada

**CTC** Claude Tessier Insect collection (private collection), Cap-Rouge, Quebec, Canada

**CMNC** Canadian Museum of Nature, Gatineau, Quebec, Canada

**CNC**Canadian National Collection of Insects, Arachnids, and Nematodes, Agriculture and Agri-Food Canada, Ottawa, Ontario, Canada

**DEBU**University of Guelph Insect Collection, University of Guelph, Guelph, Ontario, Canada

**LFC** Laurentian Forestry Center (Natural Resources Canada), René-Martineau Insectarium, Quebec, Quebec, Canada

**LLC** Ludovic Leclerc Insect Collection (private collection), Quebec, Quebec, Canada

**NBC** Nicolas Bédard Insect Collection (private collection), Quebec, Quebec, Canada

**ORC**Ouellet-Robert Collection, Université de Montréal, Montréal, Quebec, Canada

**PBC** Pierrick Bloin Insect Collection (private collection), Quebec, Quebec, Canada

**PdTC** Pierre de Tonnancour Insect Collection (private collection), Quebec, Quebec, Canada

**RVC** Robert Vigneault Insect Collection (private collection), Oka, Quebec, Canada

**SDC** Stéphane Dumont Insect Collection (private collection), Montréal, Quebec, Canada

Canadian province and territory abbreviations:

**AB** Alberta

**BC** British Columbia

**LB** Labrador

**MB** Manitoba

**NB** New Brunswick

**NF** Newfoundland

**NS** Nova Scotia

**NT** Northwest Territories

**NU** Nunavut

**ON** Ontario

**PE** Prince Edward Island

**QC** Quebec

**SK** Saskatchewan

**YT** Yukon Territory

Most specimens from 2020–2023 were collected using various active methods, such as using an entomological aspirator and by sifting various substrates (wood chips, decaying plant matter, etc.). Many individuals were captured in different types of traps, mainly pitfall traps baited with vinegar and ethanol, but also using white tulle fabric interception traps and standard flight-interception traps with collection pans underneath. Some species were also attracted with UV light, either suspended on a white sheet or combined with a white-cross-vane. Many, mostly older records, were found after consulting several collections.

Identifications were made by using available literature (see documentation under each species) or by comparing with voucher specimens housed in the LFC or the CNC. Most specimens were dissected, and their genitalia were mounted in Canada balsam or Euparal, on a microslide with the specimens. Specimens requiring confirmation were validated with external expertise, with detailed pictures or through physical examination. The illustrations were made using a Canon EOS 90D camera with a Canon MP-E 65mm f/2.8 1–5× lens, mounted on Cognisys Stackshot Macro-Rail. The images were processed and stacked using Helicon Focus, and final adjustments were made using Adobe Lightroom/Photoshop.

## ﻿New provincial and Canadian records

Adventive species are indicated with an asterisk (*) after the name. Only examined specimens deposited in private or public collections are reported under specimen data. The occurrence records from various websites, such as iNaturalist or BugGuide, are reported as “Internet data”, but only if the pictures were detailed enough for confident identification. The distribution of each species in Canada is based on the most recent available work, with new territory records placed in bold.

Label data are provided in chronological order for every species within each regional county municipality (MRC). Some data were translated from French to English, and various details known but not necessarily appearing on the labels (e.g., current MRC, GPS coordinates, collecting technique, general habitat, etc.) have been added. Each recent record follows the format: **Country: PROVINCE – County/Regional county municipality**, City [more precise location when necessary](GPS points), date of collecting, collector(s), collection method (number of specimen(s), collection abbreviation in which they are deposited). For older specimens, labels were reported verbatim, since data were frequently incomplete or imprecise.

**Family Staphylinidae Latreille, 1802**

**Subfamily Steninae MacLeay, 1825**

### ﻿ Stenus (Stenus) colon

Taxon classificationAnimaliaColeopteraStaphylinidae

Say, 1831

D83CD1FE-76B4-57EE-B264-23285BCAED2B

Fig. 1

#### Note.

This species was previously only known to reach Ontario in Canada (Bousquet et al. 2013). It can be separated from the other *Stenus* species by the wide reniform macula on each elytron, pale femora, usually with dark band at midlength, and the head broader than the elytra. In the northeast, it is unmistakable and does not resemble any other known species. It resembles to the more southerly distributed *Stenusrenifer* Lec., from which it can be readily distinguished by its larger size, broader elytra, and denser abdominal punctuation.

**Figures 1, 2.**
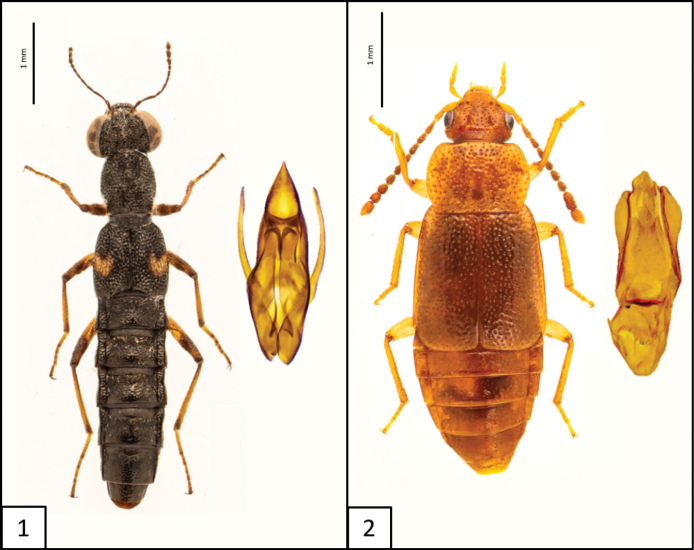
Habitus and aedeagus of **1***Stenuscolon* Say, 1831, aedeagus dorsal view **2***Arpediumschwarzi* Fauvel, 1878, aedeagus ventral view.

#### Specimen data.

**Canada: QUEBEC – MRC de Memphremagog**, Potton (45.0162, -72.4344), 20.VII-5.VIII.2022, N. Bédard, pitfall trap on a sandy river shore (1, NBC). – **Ville de Québec, Cap-Rouge** (46.7543, -71.3464), 29.VI.2023, C. Tessier, on a river bank (3, CTC). – **Ville de Lévis**, St-Nicolas (46.6902, -71.3120), 29.IX.2009, C. Tessier, sifting grass pile near a wetland (1, CTC).

#### Distribution in Canada.

ON, **QC** (Bousquet et al. 2013) - **New to Quebec**.

**Subfamily Euaesthetinae Thomson, 1859**

### ﻿ Euaesthetus similis

Taxon classificationAnimaliaColeopteraStaphylinidae

Casey, 1884

7A7081EF-BD55-5EDB-AE57-C3A23DA2EC2D

#### Note.

See Puthz (2014) for identification and illustrations. The available collection data (Puthz 2014) indicate that this species is predominantly found near water, inhabiting wetlands and areas along rivers. One specimen was collected in a muskrat nest. Less often, the species has also been collected in drier microhabitats including an alvar and cotton fields, although both of these habitats may experience flooding during heavy rains. Males can be easily recognized among other *Euaesthetus* by their strongly iridescent elytra and bifurcate parameres of the aedeagus (Puthz 2014).

#### Specimen data.

**Canada: QUEBEC – MRC de-la-Vallée-du-Richelieu**, Carignan (45.475882, -73.274623), 6.V.2022, N. Bédard, sifting river debris (1, NBC). – **MRC de Memphrémagog**, Potton (45.0259, -72.4279), 5.VIII.2022, L. Leclerc, pitfall trap baited with apple cider vinegar (2, LLC).

#### Distribution in Canada.

ON, **QC**, NB (Puthz, 2014) - **New to Quebec**.

**Subfamily Omaliinae MacLeay, 1825**

### ﻿ Arpedium schwarzi

Taxon classificationAnimaliaColeopteraStaphylinidae

Fauvel, 1878

1CFCCCEF-48BA-537D-B9F0-4FCD36F2586C

Fig. 2

#### Note.

See Campbell (1984) for identification. *Arpediumschwarzi* was previously recorded in Ontario, Canada, only from specimens collected at the hedgerow edges of soybean fields (Brunke et al. 2014, see supplementary material). Vouchers were deposited in DEBU, but the specimen data were not published. They are provided below, along with a new record from Quebec.

#### Specimen data.

**Canada: ONTARIO - Huron Co.**, Auburn (43.729, -81.528), 23.XI.2009, A. Brunke, forested hedgerow beside soybean field, pitfall (1, DEBU); Auburn (43.745, -81.508), 27.X.2010, forested hedgerow beside soybean field, near river, pitfall (1, DEBU); Auburn (43.745, -81.514), 10.XI.2010, forest hedgerow beside soybean field, pitfall (1, DEBU); Brucefield (43.509, -81.528), 23.XI.2009, A. Brunke, hedgerow near ditch, pitfall (3, DEBU). **QUEBEC - MRC de Memphrémagog**, Magog (45.281547, -72.171752), 25.V.2023, P. Bloin, sifted from *Sphagnum* moss in a bog (1, PBC).

#### Distribution in Canada.

ON, **QC** (Bousquet et al. 2013) - **New to Quebec, supporting data for Ontario**.

### ﻿ Phyllodrepa punctiventris

Taxon classificationAnimaliaColeopteraStaphylinidae

(Fauvel, 1878)

56FCCE26-9B29-5EAC-B648-E2E5157E95F2

Fig. 3

#### Note.

**Figures 3, 4.**
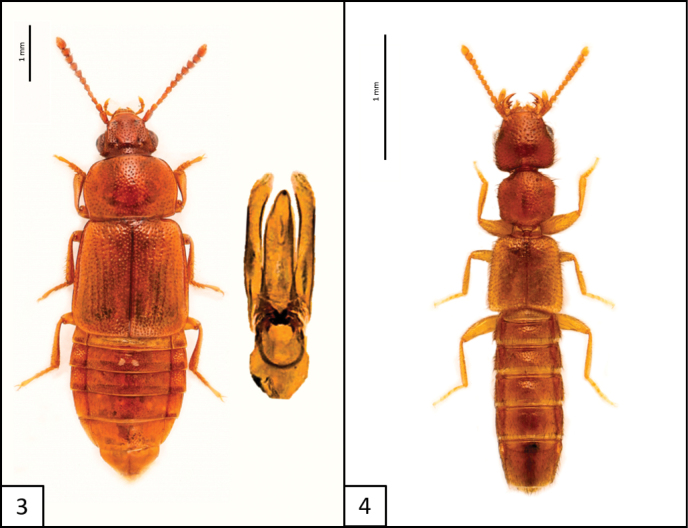
Habitus and aedeagus of **3***Phyllodrepapunctiventris* (Fauvel, 1878), aedeagus ventral view **4***Hypomedondebilicornis* (Wollaston, 1857), habitus only.

*Phyllodrepapunctiventris* is easily distinguished from other species in eastern North America by the entirely pale body. In the case of teneral specimens, it can be recognized by the pronotum with microsculpture of transverse waves across the entire disc, elytra without scratch-like sculpture between the punctures and elytral punctures in clear longitudinal rows. The parameres of the aedeagus are also distinctive (Fig. 3). Little has been published about the species’ microhabitat preferences, but it may live in bird nests or tree-holes, as one of the Quebec specimens was collected in a canopy trap and one specimen from Washington DC (CNC) was collected from an oak tree-hole. We are not aware of previously published specimen data for Ontario, so the data are provided below.

#### Specimen data.

**Canada: ONTARIO – Chatham-Kent Region**, Rondeau Provincial Park, Nature Centre, at blacklight at edge of forest, 31.V.1985, L. LeSage (1, CNC). **QUEBEC – MRC des Deux-Montagnes**, Parc National d’Oka (45.4767, -74.0537), 27.V.2002, R. Vigneault [Collected with permit] (1, RVC); Same general locality and collector but 20.V.2017 (1, RVC); Same general locality and collector but 2.V.2018, white tulle fabric interception trap in a compost site (1, LLC). – **MRC du Haut-St-Laurent**, Havelock (45.026750, -73.800528), 3–18.VI.2023, N. Bédard, Canopy cross-vane trap with fermentation bait (1, NBC).

#### Distribution in Canada.

ON, **QC** (Bousquet et al. 2013) - **New to Quebec, supporting data for Ontario**.

**Subfamily Paederinae Fleming, 1821**

### ﻿ Scopaeus (Scopaeus) minutus

Taxon classificationAnimaliaColeopteraStaphylinidae

Erichson, 1840*

824FA5AF-0569-5960-8154-C17D3803F605

#### Note.

See Brunke and Marshall (2011) for illustrations and identification. This adventive species was first reported from Montreal, Quebec, Canada, by Frisch et al. (2002) without presenting precise occurrences or vouchers. Additional data were provided by Brunke and Marshall (2011) for Ontario and by Webster et al. (2016) for New Brunswick, and we here provide the first distribution data for Quebec.

#### Specimen data.

**Canada: QUEBEC - Ville de Lévis**, Saint-Nicolas (46.6902, -71.3120), 14.V.2022, L. Leclerc, sifted from wood debris and *Sphagnum* sp. (1, LLC). - **Ville de Québec**, St-Augustin-de-Desmaures (46.7371, -71.4122), 6.V.2023, N. Bédard, sifting moss on a disturbed field (3, NBC). - **MRC de Portneuf**, Pont-Rouge (46.7543, -71.7183), 1.VIII.2022, L. Leclerc, ultraviolet cross-vane panel trap (1, LLC); same locality except 22.IV.2023, L. Leclerc, by sifting *Betula* and *Populus* leaf litter in a sandpit (2, LLC).

#### Distribution in Canada.

ON, **QC**, NB (Webster et al. 2016) - **Supporting data for Quebec**.

### ﻿ Hypomedon debilicornis

Taxon classificationAnimaliaColeopteraStaphylinidae

(Wollaston, 1857)*

0D47F57C-C87B-52C7-9EFF-B3A457D8722B

Fig. 4

#### Note.

As reported by Schülke and Smetana (2015), this cosmopolitan species has been recorded from Nearctic, Neotropical, Palearctic, Oriental, and Australian regions. Notably, this species exhibits parthenogenesis wherein females evolved without sexual reproduction, a factor that is believed to have facilitated its spread (Owen and Allen 2000). The species can be distinguished from other Medonina in eastern Canada by its pale body, small but protruding eyes, transverse subapical antennomeres, smooth lateral pronotal margins and lack of velvety appressed pubescence on the forebody. We here extend its distribution northward and newly report the species from Quebec and Canada.

#### Specimen data.

**Canada: QUEBEC - MRC Marguerite-D’Youville**, Saint-Amable (45.6431, -73.3341), 31.VIII.2023, L. Leclerc, by sifting wood chips heap (2, CNC; 5, LLC; 1, PBC; 1, NBC).

#### Distribution in Canada.

QC - **New to Canada and Quebec**.

### ﻿ Sunius melanocephalus

Taxon classificationAnimaliaColeopteraStaphylinidae

(Fabricius, 1792)*

AB6FEFC6-CCD5-5DC9-B8F9-09FE225EA129

#### Note.

See Assing (1995, 2008) and Brunke and Marshall (2011) for identification and illustrations. As in *Scopaeusminutus*, no voucher data were provided with the first Quebec record (Campbell and Davies 1991). The species was recorded for the first time in North America by Hoebeke (1991), and Brunke and Marshall (2011) added records for Ontario. We here provide supporting data for Quebec, including those for the original record, and the oldest available Canadian records for the species thus far. The species has occurred in North America since at least 1924 (Hoebeke 1991).

#### Specimen data.

**Canada: ONTARIO - Ottawa Reg.**, Kinburn, 10.X.1967, J.M. Campbell & A. Smetana, ex. nest of *Microtuspennsylvanicus* (1, CNC); Ottawa [Shirley’s Bay], 2.V.1979, A. & Z. Smetana (2, CNC); Fitzroy Provincial Park, 2–3.V.1979, A.&Z. Smetana (1, CNC); Kanata, 25.IV.1969, A. Smetana (1, CNC); Ottawa, 12.IV.1959, J.E.H. Martin (2, CNC). - **Toronto Reg.**, Toronto [Islington], 24.VIII.1990, S. Snäll (1, CNC); Toronto, 12.IX.1990, S. Snäll, lakeshore (1, CNC). **QUEBEC - Parc de la Gatineau**, King Mountain, 20.IV.1968, A. Smetana (1, CNC). - **MRC de-la-Vallée-du-Richelieu**, Carignan (45.475882, -73.274623), 6.V.2022, N. Bédard, Sifting grass pile near an urban forest (3, NBC). - **MRC de l’Île-d’Orléans**, Saint-Pierre-de-l’Île-d’Orléans (46.8813, -71.0551), 10.IX.2022, L. Leclerc, sifted from *Populus* and *Betula* leaf litter (1, LLC). - **MRC des Jardins-de-Napierville**, Sainte-Clothilde [Piège #1 carrottes] 17.VI.1985, Guy Boivin (1, CNC). - **Ville de Québec**, Sainte-Foy (46.7923, -71.2803), sifted from *Robiniapseudoacacia* leaf litter (2, LLC); Cité-Universitaire (46.7863, -71.2686), 26.IV.2023, L. Leclerc, sifted from wood chips (2, PBC).

#### Distribution in Canada.

ON, QC (Bousquet et al. 2013) - **Supporting data for Quebec**.

**Subfamily Oxyporinae**

### ﻿ Oxyporus ashei

Taxon classificationAnimaliaColeopteraStaphylinidae

Campbell, 1978

AAA8370A-8F6D-5850-B867-77919347C5F1

Fig. 5

#### Note.

See Campbell (1978) for identification. *Oxyporusashei* was described by Campbell (1978) based on four specimens from North Carolina. We newly record it here from southern Canada (QC, ON), extending its distribution far northward. This species is rarely collected but can be easily recognized by the mostly pale orange-yellow body dorsally, contrasting with the dark ventral head and thorax. Its color pattern is strikingly similar to the distantly related and common eastern species *Pseudoxyporuslateralis* (Gravenhorst, 1802) but can be distinguished by the much shorter antennae and entirely dark mandibles.

**Figure 5.**
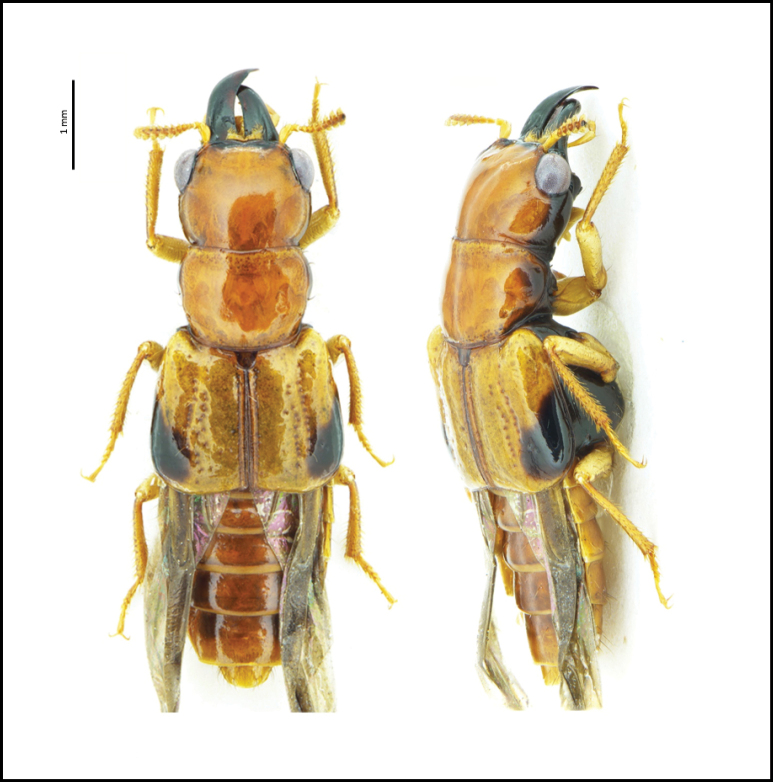
Habitus of *Oxyporusashei* Campbell, 1978, dorsal and lateral views.

#### Specimen data.

**Canada: QUEBEC – MRC des Deux-Montagnes**, Parc National d’Oka (45.472273, -74.049343), 1.VII.2018, R. Vigneault, white tulle fabric interception trap [Collected with permit] (1, RVC).

#### Internet data.

**Canada: ONTARIO**- York Co., King City (43.9635, -79.5227), 2.VIII.2021, Shuk Han (Nancy) Mak, Recorded through INaturalist (Obs.: 89701768).

#### Distribution in Canada.

ON, QC - **New to Ontario, Quebec, and Canada**.

**Subfamily Proteininae Erichson, 1839**

### ﻿ Proteinus parvulus

Taxon classificationAnimaliaColeopteraStaphylinidae

LeConte, 1863

F4F8C293-3263-5F39-AD9A-5C1EF6E395E3

#### Note.

See Webster et al. (2016) for illustrations and identification. The species was described by LeConte (1863) from “Lake Superior”. This was later corroborated by records from Ontario by Hubbard et al. (1878). More recently, Webster et al. (2016) have extended its known range by recording it from six Canadian provinces (see below). Although recognized as a transcontinental species in Canada, the distribution appeared disjunct as there have been no published records of its presence in Quebec until now. We here support this distribution with the first vouchers of the species from Quebec.

#### Specimen data.

**Canada: QUEBEC - MRC La Jacques-Cartier**, Lac-Croche (47,389896, -71,811252), 8–22.VII.2020, Christian Hébert (Canadian Forest Service), pitfall trap, projet d’aire protégée Ya’nienhonhndeh [2020-3-8822], (1, LFC); same information but (47,419579, -71,801022), 22.VII-6.VIII.2020, [2020-3-9022], (1, LFC); same information but (47,403227, -71,796046) [2020-3-8891], (1, LFC); same information but (47,259286, -71,659231), multi-directional impact trap [2020-3-8943], (1, LFC); same information but (47,371618, -71,782273) multi-directional impact trap [2020-3-8943], (2, LFC).

#### Distribution in Canada.

YT, BC, AB, SK, MBON, **QC**, NB (Webster et al. 2016) - **New to Quebec**.

**Subfamily Pselaphinae Latreille, 1802**

### ﻿ Eutyphlus schmitti

Taxon classificationAnimaliaColeopteraStaphylinidae

Raffray, 1904

DABA69C6-DF83-53D5-B6C3-F3F0E4180224

#### Note.

See Owens and Carlton (2016) for illustrations and identification. The first five specimens were found in Berlese-Tullgren extractions of forest leaf litter collected on Mont Écho in 2012 (one on 14 June, and four on 20 July). All of these specimens come from stands dominated by *Acersaccharum*, *Betulapapyrifera*, and *Fagusgrandifolia*. It represents the first record of this genus and species in Quebec and Canada. Another specimen was found in 2016 in southern Quebec, in a pitfall trap in a maple-dominated forest. *Eutyphlusschmitti* is present in mountainous regions from Quebec and New Hampshire, southward to North Carolina and westward to Ohio (Owens and Carlton 2016; present study). It was found to be particularly abundant in old-growth hardwood forests in New Hampshire (Chandler 1987).

#### Specimen data.

**Canada: QUEBEC - MRC du Brôme-Missisquoi**, Sutton (45.10389, -72.50861) 14.VI.2012, P.M. Brousseau, maple forest (1, ORC); same but 20.VII.2012 (4, ORC). - **MRC du Granit**, Lac Mégantic [148-101], 21.VII.2016, MFFP, pitfall trap 2016-0004 (1, LFC).

#### Distribution in Canada.

**QC** (Owens and Carlton 2016) - **New to Quebec and Canada**.

### ﻿ Thesium cavifrons

Taxon classificationAnimaliaColeopteraStaphylinidae

(LeConte, 1863)

6ED89AA3-76F7-55AC-993C-5ADA82C0EB66

#### Note.

See Grigarick and Schuster (1980) and Chandler (1989) for identification and illustrations. This species is the only *Thesium* in northeast North America (Chandler 2000). It can be readily distinguished from the other members of Euplectini by the carinate prosternum and the clearly separated mesocoxal cavities. In addition to the literature cited above for its identification, photos of the holotype are accessible via the MCZ website (type #27740).

#### Specimen data.

**Canada: QUEBEC - Gatineau City**, Buckingham (45°34'N, 75°28'W) 3–10.VII.2000, C. Hébert (Canadian Forest Service), Projet Verglas (1, CNC).

#### Distribution in Canada.

ON, **QC** (Bousquet et al. 2013) - **New to Quebec**.

**Subfamily Scydmaeninae Leach, 1815**

### ﻿ Euconnus (Euconnus) remiformis

Taxon classificationAnimaliaColeopteraStaphylinidae

Stephan & Chandler, 2021

C2602746-B057-5643-867D-CAFB0F531AF4

#### Note.

See Stephan et al. (2021 [2020]) for identification and illustrations. This and the following species were recently described in a Nearctic revision of the subgenusNapochus (Stephan et al. 2021 [2020]), from specimens collected in several eastern states. Both were initially described in the subgenusNapochus, but shortly after their description, Jałoszyński (2021) synonymized the subgenus with *Euconnus* s. str. *Euconnusremiformis* is mostly known from the southeastern United States, but was also reported from the northeast based on a single specimen from Maine (Stephan et al. 2021 [2020]). The present record supports a more widespread distribution in the north.

#### Specimen data.

**Canada: QUEBEC - MRC du Haut-St-Laurent**, Havelock (45.0258, -73.7993), 3–17.VII.2023, N. Bédard, Interception trap in an oak and maple forest (1, NBC).

#### Distribution in Canada.

**QC** (Stephan et al. 2021 [2020]) - **New to Quebec and Canada**.

### ﻿ Euconnus (Euconnus) separatus

Taxon classificationAnimaliaColeopteraStaphylinidae

Stephan & Chandler, 2021

4A3FCE85-9B3D-5C41-AEFE-8498549EF3A4

#### Note.

See Stephan et al. 2021 [2020]) for identification and illustrations. This species was known to occur as far north as the Upper Peninsula of Michigan, south to Florida, where it is rather common (Stephan et al. 2021 [2020]). Therefore, its presence in southern Quebec and Canada was expected and it is likely even more widespread in eastern Canada (Ontario and New Brunswick) given greater sampling effort, and modern taxonomic revision available.

#### Specimen data.

**Canada: QUEBEC - MRC du Haut-St-Laurent**, Havelock (45.0258, -73.7993), 3–17.VII.2023, N. Bédard, Interception trap in an oak and maple forest (1, NBC).

#### Distribution in Canada.

**QC** (Stephan et al. 2021 [2020]) - **New to Quebec and Canada**.

**Subfamily Scaphidiinae Latreille, 1806**

### ﻿ Baeocera inexpectata

Taxon classificationAnimaliaColeopteraStaphylinidae

Löbl & Stephan, 1993

4EBFBA93-1522-5203-A9DE-792AF54B397C

#### Note.

See Löbl and Stephan (1993) for illustrations and identification. Initially described only from Saskatchewan (Löbl and Stephan 1993), this species was recently found in New Brunswick by Webster et al. (2012e), greatly extending its range eastward. The authors suggested that it was likely to be found in the intervening territories, and this is supported by the new record from Quebec. This small species is a member of the *congener* group of species and can be easily identified by the shape and the structures of the male genitalia, with each paramere bearing a medial membranous lobe.

#### Specimen data.

**Canada: QUEBEC - MRC de Manicouagan**, Pointe-aux-Outardes (49.0943, -88.3005), 24.VI.2021, N. Bédard [#2559], handpicked in tide debris on a beach (1, NBC).

#### Distribution in Canada.

SK, **QC**, NB (Bousquet et al. 2013) - **New to Quebec**.

### ﻿ Scaphisoma americanum

Taxon classificationAnimaliaColeopteraStaphylinidae

(Löbl, 1987)

56F9105B-38D7-5737-9769-4B98B11C7164

#### Note.

See Löbl (1987) for illustrations and identification. This species was described in the genus *Caryoscapha* Ganglbauer by Löbl (1987) from various locations in eastern North America, the northernmost records being from Illinois. Despite its relatively large body, it remained overlooked in most of its range. The genus was later synonymized with *Scaphisoma* by Leschen and Löbl (2005). We report for the first time its presence in Canada, based on specimens from Quebec, Ontario, and Nova Scotia.

#### Specimen data.

**Canada: ONTARIO - Haldimand-Norfolk Reg.**, Cronmiller property [~6 km W St. Williams] (42°40'18"N, 80°29'24"W), 31.V-15.VI.2011, Brunke & Paiero, forest near vernal pools, malaise (1, DEBU); Turkey Point Provincial Park (42°41'48"N, 80°19'48"W) 19.V.2011, A. Brunke, forest site 1, Berlese leaf and log litter (1, DEBU). - **Northumberland Co.**, Peter’s Woods Provincial Nature Reserve (44°7'27"N, 78°2'21"W), 12.XI.2011, Brunke & Paiero, forest (1, DEBU); same except 6.X.2011 (2, DEBU); same except 27.VI.2011 (1, DEBU); Barr property [~ 7km NE Centreton], 1–16.VI.2011, Brunke & Paiero, field site 2, malaise, (1, DEBU). **QUEBEC - MRC de l’Île-d’Orléans**, Saint-Pierre-de-l’Île-d’Orléans (46.8772, -71.0620), 11.VI.2022, L. Leclerc, beaten from fresh *Cerioporussquamosus* (3, LLC); **Ville de Québec**, Pointe-de-Sainte-Foy (46.7506, -71.3183), 11.VI.2023, L. Leclerc, sifted from fresh *Pleurotusostreatus* (4, LLC) - **Montréal**, 1.IX.1972, E.J. Kiteley (5, CNC); same except 31.VIII.1979 (3, CNC); same except 14.VI.1983 (1, CNC). - **MRC de Bécancour**, Bécancour (Rivière Godefroy) (​​46.2977, -72.5321), 4.IX.2023, N. Bédard, sifted from *Hericiumcoralloides* (7, NBC; 1, LLC). **NOVA SCOTIA - Cape Breton Highlands National Park**, Lone Shieling, 1.VII.1983, R. Vockeroth, malaise trap (1, CNC).

#### Distribution in Canada.

ON, QC, NS - **New to Quebec, Ontario, Nova Scotia, and Canada**.

**Subfamily Staphylininae Latreille, 1802**

### ﻿ Gabrius amulius

Taxon classificationAnimaliaColeopteraStaphylinidae

Smetana, 1995

57D1A6B3-2C26-5C73-AA01-24F2F6D5A18F

Fig. 6

#### Note.

This rare species was first recorded in Canada by Brunke and Marshall (2011) from a single specimen captured in Ontario. Consistent with known habitat data, the specimen reported below was caught in a deciduous forest, and represents the first known occurrence of this species in Quebec.

**Figures 6, 7.**
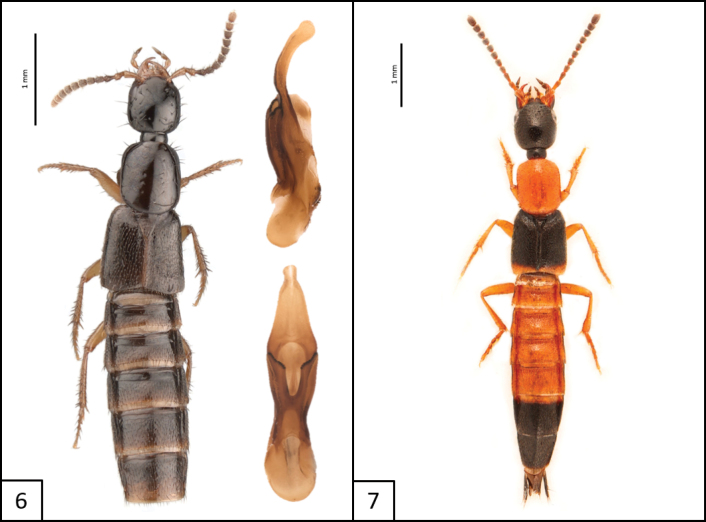
Habitus and aedeagus of **6***Gabriusamulius* Smetana, 1995, aedeagus ventral and lateral views **7***Neobisniusjucundus* (Horn, 1884), aedeagus ventral view.

#### Specimen data.

**Canada: QUEBEC - Ville de Gatineau**, Forêt Boucher (45.4208, -75.8167), 17.VI.2023, F. Génier & S. Laplante (1, CMNC).

#### Distribution in Canada.

ON, **QC** (Bousquet et al. 2013) - **New to Quebec**.

### ﻿ Neobisnius jucundus

Taxon classificationAnimaliaColeopteraStaphylinidae

(Horn, 1884)

F4CEC192-CFFB-59F4-B8FB-67519DFD3DE3

Fig. 7

#### Note.

See Frank (1981) for identification. *Neobisniusjucundus* was originally described by Horn in 1884, based on two female specimens from South Carolina. It has since been found to have a widespread presence in North America, extending into several Canadian provinces (Bousquet et al. 2013). Here we further extend its known Canadian distribution to include southern Quebec. It becomes the fifth species of the genus known from Quebec. Among the bicolored species of the genus, it can be recognized in eastern Canada by the following combination of characters: head longer than wide and with obvious microsculpture dorsally; eyes occupying ~ ⅓ of head length; elytra narrowly pale at apex (< 40%); one or more palpomeres of maxillary palpus darkened. The species can also be recognized by the distinctive shape of the aedeagus (Frank 1981).

#### Specimen data.

**Canada: QUEBEC - MRC des Deux-Montagnes**, Parc national d’Oka (45.476466, -74.054149), 11.V.2023, R. Vigneault, white tulle fabric tissue in a compost site [Collected with permit] (1, RVC). - **MRC de Memphrémagog**, Potton (45.0259, -72.4279), 5.VI.2022, L. Leclerc, N. Bédard & P. Bloin, handpicked under flood debris (1, LLC; 3, NBC; 1, PBC); same locality, except 16.VI.2022 (1, PBC); same locality except 20.VII-5.VIII.2022, pitfall trap baited with apple cider vinegar (1, LLC). - **MRC du Granit**, Saint-Augustin-de-Woburn (45.416694, -70.879500), 12.V.2022, sandy-gravelly bank of a small river littered with woody debris, N. Bédard & P. Bloin (3, NBC; 1, PBC).

#### Distribution in Canada.

BC, AB, SK, MB, ON, **QC**, NB (Bousquet et al. 2013) - **New to Quebec**.

### ﻿ Ocypus nitens

Taxon classificationAnimaliaColeopteraStaphylinidae

(Schrank, 1781)*

BB08B936-D7D9-5E60-B0B1-0E64CD4BB0DB

#### Note.

See Brunke et al. (2011) and Brunke (2016) for illustrations and identification. This large adventive species is native to Europe, the Caucasus, Iran, and Turkey (Herman 2001), and was first detected in eastern North America in Massachusetts in 1944 (Newton 1987). For more than fifty years, it apparently remained confined to a small area in New England (Newton 1987; Brunke 2016), but it has since expanded rapidly its range to Maine by 1989, Rhode Island by 1995 (Brunke et al. 2011), New York by 2010, Vermont and Ontario by 2014 (Brunke 2016) and New Brunswick by 2018 (Knopf and Gilmore 2018). Records from BugGuide were mentioned in Brunke (2016), but those from iNaturalist were not considered, and are here referred-to because they represent most of the observations available for the province of Quebec and Nova Scotia, and greatly extend its known range. The species has been known from Quebec since at least 2013 according to iNaturalist records. There were 38 INaturalist observations for the province of Quebec, 33 of which were confirmed and verified. These data (grouped here) represent a widespread area in southern Quebec, reaching its northernmost limit at the level of Montreal and Sherbrooke south to Godmanchester and Potton. We also provide physical specimen data to support the presence of *Ocypusnitens* in Quebec.

#### Specimen data.

**Canada: QUEBEC - Montréal** (45.5436, -73.6901), 31.V.2018, S. Dumont, pitfall trap (1, SDC); (45.5430 -73.6911), 13.VI.2023, handpicked under a rock (2, SDC); (45.5436, -73.6901), 20.X.2023, pitfall trap (2, SDC); 23.X.2023 (1, SDC); 12.XI.2023 (5, SDC); 16.XI.2023 (2, SDC), 17.XI.2023 (2, SDC). - **MRC de Brome-Missisquoi**, Saint-Armand (45.0221, -73.0582), 29.VII.2017, L. Leclerc, under hardwood log (1, LLC). - **MRC de Deux-Montagnes**, Parc national d’Oka, (Grande Baie, 45.4906, -74.0111), 9.X.2019, 14:00, P. de Tonnancour, climbing tree trunk [Collected with permit] (1, PdTC). - **MRC de Memphremagog**, Potton (45.0162, -72.4344), 15–29.VII.2022, N. Bédard, pitfall trap in a mixed maple forest, det.: NB (1, NBC); Stanstead-Est (45.1578, -72.0291), 28.IV.2018, S. Mailhot, caught in flight (1, LLC). - **MRC du Val-Saint-François**, Racine (45.459475, -72.161956), 15.V.2021, P. Bloin, under log of deciduous tree (1, PBC). - **MRC Les Appalaches**, Adstock (46.0049, -71.1104), 5.XI.2022, P. Bloin, sifted from moss in a balsam fir stand (1, PBC).

#### Internet data.

**Canada: NOVA SCOTIA - Annapolis Co.**, Clementsvale (44.635474, -65.566914), 19.X.2021, Alexis Orion, Recorded through INaturalist (Obs.: 99352464); Lake La Rose (44.705801, -65.440164), 20.III.2022, Ashlea Viola (@ashlea03), Recorded through INaturalist (Obs.: 109590192); Round Hill (44.769997, -65.409845), 14.VI.2022, (@spaceexplorer), Recorded through INaturalist (Obs.: 121718039). - **Lunenberg Co**., Chelsea (44.374644, -64.727725), 13.IV.2022, Heather Haughn (@hhaughn), Recorded through INaturalist (Obs.: 111330891); Chelsea (44.374808, -64.727878), 13.IX.2022, Heather Haughn (@hhaughn), Recorded through INaturalist (Obs.: 134902909); Chelsea (44.374642, -64.727708), 16.IX.2022, Heather Haughn (@hhaughn), Recorded through INaturalist (Obs.: 135278623); Conquerall (44.311425, -64.554755), 6.VII.2023, Jamie VanBuskirk (@jamievanburskirk), Recorded through INaturalist (Obs.: 171483228). - **Kings Co**., Kentville (45.076912, -64.494473), 22.V.2022, (@kmelville), Recorded through INaturalist (Obs.: 118447209); Bishopsville (45.014267, -64.275407), XII.2022, (@cricket_toadums), Recorded through INaturalist (Obs.: 144268319); Kentville (45.062484, -64.56368), 13.V.2023, Dan Casey (@dan_casey), Recorded through INaturalist (Obs.: 172019838); Casey Corner (45.01433, -64.566744), VI.2023, (@cricket_toadums), Recorded through INaturalist (Obs.: 169149071).

#### Distribution in Canada.

ON, **QC**, NB, **NS** (Brunke 2016) - **New to Quebec and Nova Scotia**.

### ﻿ Platydracus exulans

Taxon classificationAnimaliaColeopteraStaphylinidae

(Erichson, 1839)

D8F9A364-457D-5E0F-B73B-F56C97DAAA7F

#### Note.

See Brunke et al. (2011) for illustrations and identification. This native species was reported from Quebec by Downie and Arnett (1996) without any further information, but because this record was later presumed to be based on a misidentified specimen (Brunke et al. 2011), it was not reported for Quebec by Bousquet et al. (2013). In Ontario, it has been collected only twice (once with two specimens) and, moreover, 44 years apart at the same locality near the Ottawa River in the Ottawa area. The Quebec specimen was also found along the Ottawa River further east and it is not clear whether these are vagrant specimens or if there is an apparently disjunct northern population of this species.

#### Specimen data.

**Canada: QUEBEC - MRC de Vaudreuil-Soulanges**, Rigaud (45.4906, -74.2919), 3.VII.2020, N. Bédard, UV light trap (1, NBC).

#### Distribution in Canada.

ON, **QC** (Bousquet et al. 2013) - **New to Quebec**.

### ﻿ Philonthus hepaticus

Taxon classificationAnimaliaColeopteraStaphylinidae

Erichson, 1840

816D8050-A0B2-5E98-883D-754AE7B459CA

Fig. 8

#### Note.

See Smetana (1995) for identification. Majka et al. (2009b) documented a significant range extension for this *Philonthus* species, shedding light on existing distribution gaps within the eastern rove beetle fauna. While this species displays a bipartite distribution pattern in Canada, Smetana (1995) indicated that it is a transcontinental species, with its presence in Quebec highly expected. *Philonthushepaticus* has an extremely broad range in the New World and occurs south to Chile and Argentina. It has also become adventive in Australia and New Zealand (Newton 2022). This species probably also occurs in at least some parts of southern Ontario.

**Figures 8, 9.**
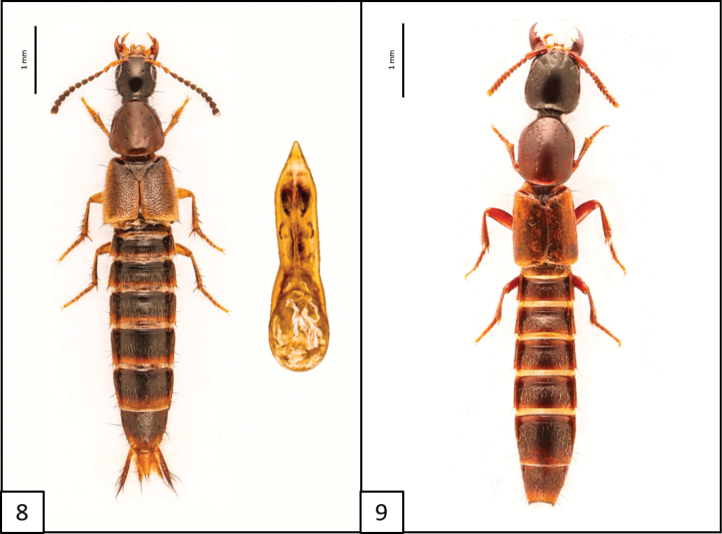
Habitus and aedeagus of **8***Philonthushepaticus* Erichson, 1840, aedeagus ventral view **9***Hypnogyragularis* (LeConte, 1880), habitus only.

#### Specimen data.

**Canada: QUEBEC - Ville de Québec**, Cité-Universitaire (46.7863, -71.2686), 30.V.2023, 18:00–21:00, L. Leclerc, white tulle fabric interception trap (1, LLC); Plaines d’Abraham (46.7950, -71.2285) 2.IX.2023, L. Leclerc, sifted from wood chips heap (4, LLC); Sainte-Foy (46.7874, -71.2914), 28.IX.2023, L. Leclerc, sifted from decaying grass heap (1, LLC); 30.IX.2023 (1, LLC); 5.X.2023 (1, LLC); 9.X.2023 (1, LLC); 11.X.2023 (1, LLC). - **MRC des Deux-Montagnes**, Parc National d’Oka (45.4767, -74.0537), 10.XI.2020, R. Vigneault, white tulle fabric interception trap in a compost site [Collected with permit] (1, RVC); 25.X.2022 (2, RVC).

#### Distribution in Canada.

BC, **QC**, NB (Bousquet et al. 2013) - **New to Quebec**.

### ﻿ Philonthus sanguinolentus

Taxon classificationAnimaliaColeopteraStaphylinidae

(Gravenhorst, 1802)*

23B5C309-8099-5FEB-B4EF-DF115FB1B403

#### Note.

See Smetana (1995) and Klimaszewski et al. (2013) for illustrations and identification. This adventive Palearctic species was initially restricted in North America to the Pacific coast (Smetana 1995), but was discovered in 2013 (collected in 2011) for the first time in Ontario (Klimaszewski et al. 2013). In 2017, a photo record of a specimen from Quebec, Canada was published on BugGuide (see “Internet data” below). We hereby support this new Quebec record with specimen data below.

#### Specimen data.

**Canada: QUEBEC - Ville de Québec**, Cap-Rouge (46.7519, -71.3069), 17.VII.2022, P. Bloin, by sweeping vegetation along the railway track (1, PBC); Sainte-Foy (46.7874, -71.2914), 23.VII.2023, L. Leclerc, sifted from a decaying grass heap (1, LLC).

#### Internet data.

**Canada: QUEBEC - MRC de la Haute-Yamaska**, Granby, 17.VIII.2017, J. Brodeur, recorded through BugGuide (https://BugGuide.net/node/view/1425701).

#### Distribution in Canada.

ON, **QC** (Bousquet et al. 2013) - **New to Quebec**.

### ﻿ Quedius cinctus

Taxon classificationAnimaliaColeopteraStaphylinidae

(Paykull, 1790)*

9664DA7D-7535-50DA-83E2-36935859D01C

#### Note.

See Majka et al. (2009a) and Smetana (1971) for illustrations and identification. Known to be present in North America since at least 1942 (Smetana 1971). This adventive species was recorded for the first time in Canada by Majka et al. (2009a) from specimens collected on carrion in New Brunswick in 2007. Across its native western Palaearctic range, this species lives mainly in decaying organic substances, very often near or directly in human settlements (Smetana 1971). Brunke and Marshall (2011) reported it from Ontario based on specimens collected from rotting *Cerioporus* (= *Polyporus*) *squamosus* in 2008.

#### Specimen data.

**Canada: QUEBEC - Ville de Québec**, Plaines d’Abraham (46.795042, -71.228484), 6.IX.2022, P. Bloin, sifted from wood chips and plant waste (2, PBC, 1, NBC); Sainte-Foy (46.7874, -71.2914), 24.X.2022, white tulle fabric interception trap (4, LLC), 5.V.2023 (1, LLC), 9.X.2023, sifted from decaying grass heap (1, LLC), 10.X.2023 (1, LLC), 14.X.2023 (1, LLC), 17.X.2023 (1, LLC) ; Cité-Universitaire (46.7863, -71.2686), 15.X.2022, L. Leclerc, N. Bédard & P. Bloin, sifted from fresh wood chips pile (1, LLC), 23.X.2022 (2, LLC), 25.X.2022 (4, LLC; 4, NBC; 4, PBC), 4.XI.2022, white tulle fabric interception trap (4, LLC). - **MRC de Deux-Montagnes**, Oka (45.4993, -74.0203), 3.IV.2020, R. Vigneault, white tulle fabric interception trap in a compost site (1, RVC); Parc national d’Oka (45.4767, -74.0537), 22.III.2021, R. Vigneault, white tulle fabric interception trap in a compost site [Collected with permit] (1, RVC); 12.X.2022, P. de Tonnancour and R. Vigneault, white tulle fabric interception trap [Collected with permit] (2, PdTC; 1, RVC). - **MRC de l’Île-d’Orléans**, Saint-Pierre-de-l’Île-d’Orléans (46.8809, -71.0636), 5.X.2023, 16:00–18:00, L. Leclerc, white tulle fabric interception trap (1, LLC). - **MRC de Vaudreuil-Soulanges**, Terrasse-Vaudreuil (45.3923, -73.9922), 26-IX-2011, P. de Tonnancour, fermented cantaloup (1, PdTC); 27.IX.2018, P. de Tonnancour, attracted to a compost heap (1, PdTC); 7.X.2021, P. de Tonnancour, composted grass clippings (1, PdTC); 30.IX.2023, 15:00–17:00, P. de Tonnancour, white tulle fabric interception trap (1, PdTC). - **Ville de Gatineau**, Aylmer [Ouest Forêt Boucher], 15.IV.2010, V. Théberge & L. LeSage, Berlese of porcupine dung in a hollow base of a large maple tree, in a mixed forest (1, CNC); same except 6.IV.2010 (4, CNC).

#### Distribution in Canada.

ON, **QC**, NB (Bousquet et al. 2013) - **New to Quebec**.

### ﻿ Hypnogyra gularis

Taxon classificationAnimaliaColeopteraStaphylinidae

(LeConte, 1880)

B80BBDF1-DE36-58B6-8FB1-19353C178109

Fig. 9

#### Note.

See Smetana (1982) for identification. This species has been previously reported from New Brunswick (Webster et al. 2012h) and Ontario (Bousquet et al. 2013). Not much has been reported about its biology, though Smetana (1982) suspected that it prefers microhabitats similar to those of the Central European species, *H.angularis* (Ganglbauer, 1895), which is associated with tree-holes and similar microhabitats, and often cohabitates with wood-nesting ants. One of us (AJB) has repeatedly collected series of this species in tree-holes (oaks, beech, sugar maple) in Ontario, confirming the hypothesis of Smetana (1982).

#### Specimen data.

**Canada: QUEBEC – Ville de Québec**, Sainte-Foy (46.7874, -71.2914), 17.V.2021, L. Leclerc, white tulle fabric interception trap (1, LLC). – **MRC des Deux-Montagnes**, Parc National d’Oka (45.4767, -74.0537), 5.V.2019, R. Vigneault, white tulle fabric interception trap in a compost site [Collected with permit] (1, LLC); 20.V.2019 (11, LLC); La Grande Baie (45.4927, -74.0056), 10.V.2022, 15:00–16:00, P. de Tonnancour, white tulle fabric interception trap in a sugar maple stand [Collected with permit] (1, PdTC). – **MRC du Haut-St-Laurent**, Havelock (45.026750, -73.800528), 3–18.VI.2023, N. Bédard, Canopy cross-vane trap with fermentation bait (1, NBC); Same locality and collector but 18.VI-3.VII.2023, interception trap in a maple and oak forest (1, NBC).

#### Distribution in Canada.

ON, **QC**, NB (Bousquet et al. 2013) - **New to Quebec**.

### ﻿ Gauropterus fulgidus

Taxon classificationAnimaliaColeopteraStaphylinidae

(Fabricius, 1787)*

A92D0A63-0E49-564A-BC14-E44911D4996C

Fig. 10

#### Note.

See Smetana (1982) for identification. This very characteristic and large xantholinine beetle was accidentally introduced to North America from Europe in the 19^th^ century and is now found in both the western and eastern parts of North America (Smetana 1982). We report here the first occurrences of this species in the province of Quebec.

**Figures 10, 11.**
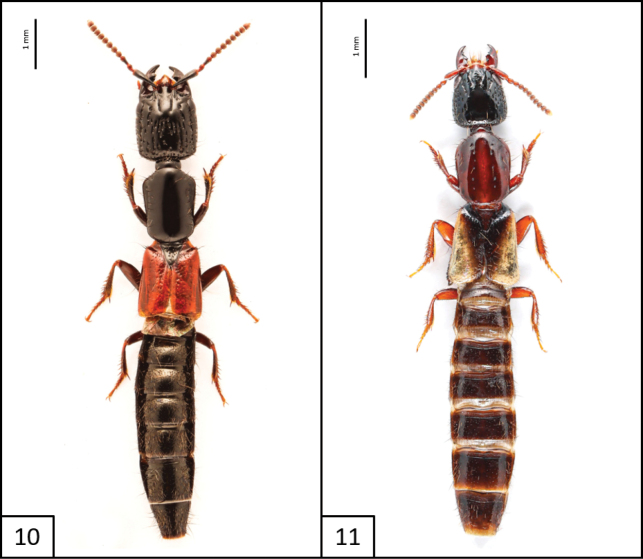
Habitus of **10***Gauropterusfulgidus* (Fabricius, 1787) **11***Phacophalluspallidipennis* (Motschulsky, 1858).

#### Specimen data.

**Canada: QUEBEC – MRC des Deux-Montagnes**, Parc National d’Oka (45.476714, -74.053690), 27.V.2023, R. Vigneault, white tulle fabric interception trap in a compost site [Collected with permit] (1, NBC). – **Ville de Québec**, Beauport (46.9421, -71.1987), 21.V.2021, N. Bédard, white tulle fabric interception trap (1, NBC); Sainte-Foy (46.7921, -71.2806), 15.X.2022, L. Leclerc, sifted from dried vegetal debris (2, LLC); Cité-Universitaire (46.7863, -71.2686), 26.X.2022, P. Bloin, sifted from wood chips (2, PBC).

#### Distribution in Canada.

ON, **QC** (Bousquet et al. 2013) - **New to Quebec**.

### ﻿ Phacophallus pallidipennis

Taxon classificationAnimaliaColeopteraStaphylinidae

(Motschulsky, 1858)*

0FCC308D-1FA4-5502-9ECA-1458A81B89B9

Fig. 11

#### Note.

See Smetana (1982) for identification. This Oriental species is adventive in Europe, North America, Africa and the Australian region, and was initially identified in North America in 1904 along the western coast. Since then, it has been observed in various locations across the continent. The first detection in the eastern part of North America was in New York in 1931 (Smetana 1982). The records given below represent its first detection in Canada and are the northernmost known. It is generally a species that is more commonly found in warmer and southern regions of North America (Smetana 1982). It was previously reported as *Phacophallustricolor* in most recent works (including Smetana 1982), but was synonymized with *Phacophalluspallidipennis* by Bordoni (2002).

#### Specimen data.

**Canada: QUEBEC – MRC de Marguerite-D’Youville**, Varennes, C. Chantal, 8.IX.2020 (1), 11.IX.2020 (2), 17.X.2020 (2), 23.X.2020 (4), 17.VIII.2021 (2), sifting dead grass (13, CCC). – **Ville de Québec**, Cité-Universitaire (46.7861, -71.2687), 23.X.2022, L. Leclerc, white tulle fabric interception trap (2, LLC); same locality except 26.X.2022, N. Bédard, sifting decomposing wood chips (1, NBC); same locality and method except 28.X.2022 (3, PBC).

#### Distribution in Canada.

QC. - **New to Quebec and Canada**.

### ﻿ Xantholinus linearis

Taxon classificationAnimaliaColeopteraStaphylinidae

(Olivier, 1795)*

E5223E79-0C94-52D1-AC9D-0C5E4B108EBE

#### Note.

See Brunke and Majka (2010) for illustrations and identification. This introduced species was recently detected in Quebec based on specimens captured by the authors. However, in the future, by inspecting older or uncurated material in collections, older specimens may be found. This species has been present in the maritime provinces of Canada and eastern North America since at least 1949 (Brunke and Majka 2010) but was first detected in North America in 1930 (British Columbia) (Smetana 1982).

#### Specimen data.

**Canada: QUEBEC – MRC du Granit**, Stratford (45.760829, -71.345770), 18.VIII.2023, N. Bédard, Handpicked in a parking lot (1, NBC). – **MRC La-Côte-de-Beaupré**, Saint-Joachim (47.0669, -70.8014), 15.X.2022, P. Bloin, flying on a warm fall day (1, PBC). – **Ville de Lévis**, Saint-Romuald (46.7390, -71.2615), 29.IV.2023, L. Leclerc, sifted from *Quercus* and *Acer* leaf litter (1, LLC).

#### Distribution in Canada.

BC, AB, ON, **QC**, NB, NS, PE, NF (Bousquet et al. 2013) - **New to Quebec**.

**Subfamily Tachyporinae MacLeay, 1825**

### ﻿ Sepedophilus basalis

Taxon classificationAnimaliaColeopteraStaphylinidae

(Erichson, 1839)

E557E8F9-AD49-51F4-B52E-070D5A03397A

Fig. 12

#### Note.

See Campbell (1976) for identification. *Sepedophilusbasalis* is listed as occurring in Ontario by Bousquet et al. (2013) and Ontario and Quebec by Newton (2022). However, we have corresponded with these authors and could not determine a published voucher-based source for the records. It is possible the Quebec record came from the historical account by Provancher (1877: 243), although misidentifications with other species were common before the modern revision by Campbell (1976). The inclusion of this species in the Ontario fauna likely came from unpublished data of specimens deposited in the CNC. We here provide voucher data for *Sepedophilusbasalis*, which occurs in Canada, broadly from southern Ontario to southern Quebec.

**Figure 12.**
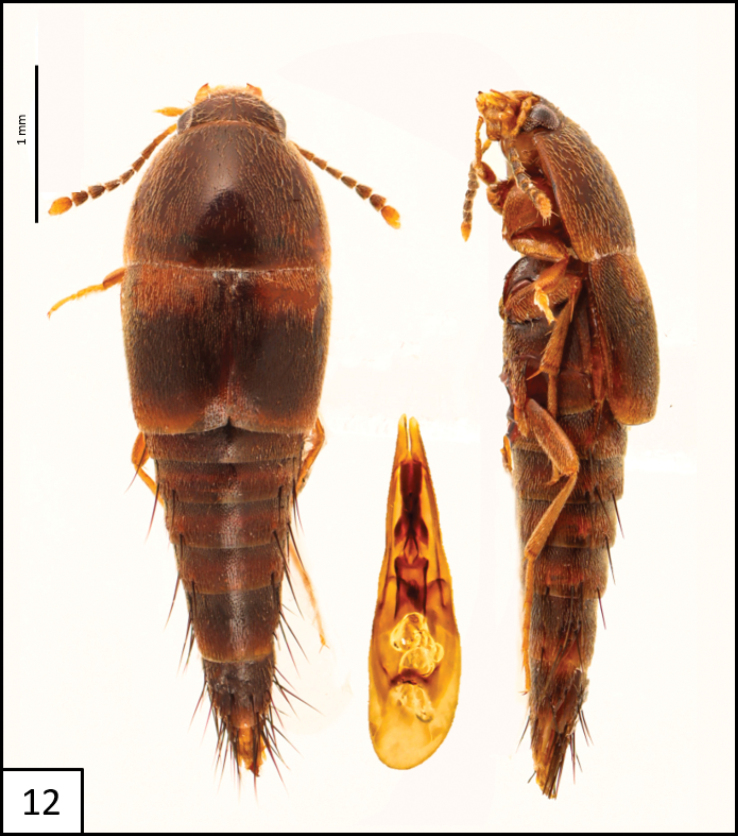
Habitus of *Sepedophilusbasalis* (Erichson, 1839) dorsal and lateral views, and aedeagus (ventral view).

#### Specimen data.

**Canada: QUEBEC – MRC de Bécancour**, Bécancour (Rivière Godefroy) (46.2977, -72.5301), 25.V.2022, N. Bédard, sifted from debris near a river (3, NBC). – **MRC des Deux-Montagnes**, Parc National d’Oka (45.472273, -74.049343), 11.V.2023, R. Vigneault, white tulle fabric interception trap in a compost site [Collected with permit] (1, NBC); same but 13.V.2023 (1, NBC). **ONTARIO – Chatham-Kent Reg.**, Rondeau Provincial Park,11–25.V.1985, L. LeSage & A. Woodliffe, intercept trap 4, white pine stand (1, CNC); same except 2–13.VII.1985, intercept trap in maple beech forest (2, CNC); Rondeau Provincial Park, 25–28.V.1985, L. LeSage & A. Smetana, intercept trap in maple beech forest (1, CNC); same except 14.VI.-2.VII.1985 (1, CNC); Rondeau Provincial Park, 14.VI.-2.VII.1985, L. LeSage & D.M. Wood, intercept trap in maple beech forest (1, CNC); Rondeau Provincial Park, [South Point Trail], 31.V.1985, A. Smetana (5, CNC); Rondeau Provincial Park, [N end South Point Trail], 3.VI.1985, A. Davies & J.M. Campbell, under bark of fallen tree (5, CNC); Rondeau Provincial Park [Spicebush Trail], 4.VI.1985, A. Davies & J.M. Campbell, sifting mushrooms and litter (4, CNC); same except under bark of fallen tree (2, CNC); Rondeau Provincial Park, [Harrison Trail], 30.V.1985, A. Smetana (2, CNC); Rondeau Provincial Park [South Point], 2.VI.1985, A. Davies & J.M. Campbell, moss on log in pond (1, CNC); Rondeau Provincial Park [Tulip Tree Trail], 5.VI.1985, A. Davies & J.M. Campbell, sifting beech and maple litter near water (1, CNC); **Elgin Co.**, J.F. Pearce Park, 5.VI.1981, L. LeSage (1, CNC). – **Halton Reg.**, Milton, 21–30.VIII.1981, M. Sanborne (1, CNC). – **Leeds and Grenville Co.**, 2 km SE Spencerville, 30.IV.1979, A.& Z. Smetana (1, CNC). – **Ottawa Reg.**, 5 km NW South March, 24.IV.1979, A.& Z. Smetana (1, CNC).

#### Distribution in Canada.

ON, **QC - New to Quebec, supporting data for Ontario**.

## ﻿Discussion

Distribution data of 27 species of rove beetles (excluding Aleocharinae) are provided for the Province of Quebec, 25 of which are new records, increasing the total number of staphylinid species in Quebec to 863 (Bédard, unpublished database). Approximately one-third of the newly recorded species (10 out of 27) are considered adventive in North America. Notably, these adventive species were predominantly found in human-disturbed habitats, including compost heaps and wood chip piles. These man-made habitats offer favorable conditions for introduced species as they tend to be warmer and to have more stable temperatures than the surrounding environments as a result of the heat generated by decomposition. This phenomenon was observed in several beetle families in Europe, where warm-loving species tended to thrive farther north in compost compared to other microhabitats (Ødegaard and Tømmerås 2000). Some of the species we report here, including *Gauropterusfulgidus*, *Phacophalluspallidipennis*, *Philonthussanguinolentus* and *Hypomedondebilicornis* appear to follow this principle.

Moreover, sifting these different substrates also revealed new records of species from largely tropical genera, such as *Echiaster* Erichson, 1839 and *Atanygnathus* Jakobson, 1909. These species could not be identified but are not among the described North American species. They could not be treated in this paper because the genera are unrevised across most of their distribution. The occurrence of these species in Canada may be attributed to the transport of “contaminated” plant material (Klimaszewski and Brunke 2018). In the case of many species in the present paper, large accumulations of wood chips could be considered refugia for these species, allowing them to survive harsh winters and expand further into synanthropic and natural environments (Ødegaard and Tømmerås 2000).

The detection of the above rove beetle species in Quebec is likely due to a very recent intensification of sampling effort in the province, combined with the use of alternative collection methods. *Hypnogyragularis*, *Oxyporusashei*, and *Quediuscinctus* were mainly collected using the white tulle fabric interception trap, which seems to effectively capture small, cryptic species and those that are highly local and frequently disperse to patchy or ephemeral microhabitats (de Tonnancour et al. 2017). Notably, while Quebec was included in several broad taxonomic works (e.g., Klimaszewski et al. 2018, 2021) and has been the site of several forestry studies (Paquin and Dupérré 2001; Klimaszewski et al. 2007), the province has never been the focus of recent faunistic or revisionary research on rove beetles. The present paper has begun to narrow this knowledge gap and with continued sampling and taxonomic effort, we hope to better understand the true diversity of Staphylinidae in Quebec.

## Supplementary Material

XML Treatment for Stenus (Stenus) colon

XML Treatment for Euaesthetus similis

XML Treatment for Arpedium schwarzi

XML Treatment for Phyllodrepa punctiventris

XML Treatment for Scopaeus (Scopaeus) minutus

XML Treatment for Hypomedon debilicornis

XML Treatment for Sunius melanocephalus

XML Treatment for Oxyporus ashei

XML Treatment for Proteinus parvulus

XML Treatment for Eutyphlus schmitti

XML Treatment for Thesium cavifrons

XML Treatment for Euconnus (Euconnus) remiformis

XML Treatment for Euconnus (Euconnus) separatus

XML Treatment for Baeocera inexpectata

XML Treatment for Scaphisoma americanum

XML Treatment for Gabrius amulius

XML Treatment for Neobisnius jucundus

XML Treatment for Ocypus nitens

XML Treatment for Platydracus exulans

XML Treatment for Philonthus hepaticus

XML Treatment for Philonthus sanguinolentus

XML Treatment for Quedius cinctus

XML Treatment for Hypnogyra gularis

XML Treatment for Gauropterus fulgidus

XML Treatment for Phacophallus pallidipennis

XML Treatment for Xantholinus linearis

XML Treatment for Sepedophilus basalis

## ﻿Additional information

### Author ORCIDs

Nicolas Bédard https://orcid.org/0009-0004-7649-100X

Adam Brunke https://orcid.org/0000-0003-1158-936X

Pierrick Bloin https://orcid.org/0009-0009-3260-9017

Ludovic Leclerc https://orcid.org/0009-0006-2381-9627

### Data availability

All of the data that support the findings of this study are available in the main text.
